# Diagnostic Performance of the PalmScan VF2000 Virtual Reality Visual Field Analyzer for Identification and Classification of Glaucoma

**DOI:** 10.18502/jovr.v17i1.10168

**Published:** 2022-01-21

**Authors:** Vijay Shetty, Prachi Sankhe, Suhas S Haldipurkar, Tanvi Haldipurkar, Rita Dhamankar, Priyanka Kashelkar, Dhruven Shah, Paresh Mhatre, Maninder Singh Setia

**Affiliations:** ^1^Laxmi Eye Institute, Panvel, Maharashtra, India

**Keywords:** Glaucoma; Sensitivity, Specificity, Test Properties, Virtual Reality Perimetry

## Abstract

**Purpose:**

To evaluate the diagnostic test properties of the Palm Scan VF2000Ⓡ Virtual Reality Visual Field Analyzer for diagnosis and classification of the severity of glaucoma.

**Methods:**

This study was a prospective cross-sectional analysis of 166 eyes from 97 participants. All of them were examined by the HumphreyⓇ Field Analyzer (used as the gold standard) and the Palm Scan VF 2000Ⓡ Virtual Reality Visual Field Analyzer on the same day by the same examiner. We estimated the kappa statistic (including 95% confidence interval [CI]) as a measure of agreement between these two methods. The diagnostic test properties were assessed using sensitivity, specificity, positive predictive value (PPV), and negative predictive value (NPV).

**Results:**

The sensitivity, specificity, PPV, and NPV for the Virtual Reality Visual Field Analyzer for the classification of individuals as glaucoma/non-glaucoma was 100%. The general agreement for the classification of glaucoma between these two instruments was 0.63 (95% CI: 0.56-0.78). The agreement for mild glaucoma was 0.76 (95% CI: 0.61-0.92), for moderate glaucoma was 0.37 (0.14-0.60), and for severe glaucoma was 0.70 (95% CI: 0.55-0.85). About 28% of moderate glaucoma cases were misclassified as mild and 17% were misclassified as severe by the virtual reality visual field analyzer. Furthermore, 20% of severe cases were misclassified as moderate by this instrument.

**Conclusion:**

The instrument is 100% sensitive and specific in detection of glaucoma. However, among patients with glaucoma, there is a relatively high proportion of misclassification of severity of glaucoma. Thus, although useful for screening of glaucoma, it cannot replace the HumphreyⓇ Field Analyzer for the clinical management in its current form.

##  INTRODUCTION

Glaucoma, the second most common cause of vision loss in the world, is an important cause of blindness in India.^[1,2,3]^ About 6.48 million people were estimated to have primary open-angle glaucoma in India.^[3]^ It may be rightly termed as “the silent disease”, as it causes bilateral, painless, and progressive vision loss.^[4]^ One of the instruments used for the diagnosis and management of glaucoma, the HumphreyⓇ Field Analyzer (Zeiss/USA) (HFA), is an automated perimeter and is well-known to ophthalmologists and optometrists. It is considered to be accurate, reliable, and a trusted method to detect the visual field defects of patients.^[5]^ However, like other devices, HFA also has certain disadvantages and limitations. It is big and bulky, non-portable, demands a dark room, time-consuming, and may be difficult for patients with neck problems, old age, children, or those with any disability where it is difficult to keep their heads in a fixed slot to maintain good fixation.^[6]^

The Palm Scan VF2000Ⓡ (MMD/USA) is a virtual reality (VR)-based visual field analyzer developed to measure the patient's visual field defect. It is a battery-operated portable device. The VF2000 consists of a system with three main sections connected to each other by a wireless mechanism. These three major components are: (1) the VR goggles worn by the patients; (2) the controller device operated by the healthcare staff who sets the testing strategy, technical parameters, and monitors the entire test; and (3) the clicker, which will be clicked by the patient when visualizing the stimulus.^[6]^ There is a classic perimeter bowl in HFA whereas the Palm Scan VF 2000Ⓡ VR Visual Field Analyzer has VR goggles. However, the VR perimetry has algorithms in place to make the stimuli appear on the retina as if they have been projected from the classic perimeter bowl.^[7]^ The entire VR perimetry system fits in a small portable unit and it does not require a dedicated dark room or the fellow eye to be occluded. Furthermore, the VF2000 perimeter may be a more practical device for examining the visual fields in children, as well as in patients who are unable to perform HFA testing such as those with disabilities, those in nursing homes, and those who are hospitalized.^[7]^

Although, there are apparent advantages of the VF2000, it is also important to evaluate its accuracy in diagnosis and classification of glaucoma. Previous studies have shown that there is a correlation between the tablet-based visual field assessment and HFA; however, they have not discussed the performance of these instruments in the classification of the severity of glaucoma.^[7,8]^ With this background, we designed the present study to evaluate the diagnostic test properties of the Palm Scan VF2000Ⓡ VR visual field analyzer for the diagnosis of glaucoma and the classification of the severity of glaucoma. We compared the agreement for diagnosis and classification of glaucoma between VR perimetry and HFA.

##  METHODS

The present study was a prospective cross-sectional analysis of 166 eyes from 97 participants.

### Study Site

The study was conducted at the Laxmi Eye Hospital, a tertiary eye care center situated at a distance of about 50 km from Mumbai, India. The study was approved by the Ethics Committee at LEI for primary data analysis.

### Study Population 

We recruited 97 consenting patients who presented to the center. Of these, 57 individuals (86 eyes) had glaucoma and 40 (80 eyes) did not. The inclusion criteria for the glaucoma group were: (1) aged between 20 and 65 years; (2) those classified as glaucoma based on the Anderson criteria^[9]^ – three non-edge points on the pattern deviation map, pattern standard deviation (PSD), and glaucoma hemifield test along with an intraocular pressure (IOP) of ≥ 20; and (3) those consenting for the study. The inclusion criteria for the non-glaucoma group were: (1) aged between 20 and 65 years; (2) those who were negative for all three parameters in the aforementioned criteria with an IOP of < 20; and (3) those consenting for the study. The exclusion criteria were: (1) those with visual acuity < 6/60 and (2) any other coexisting ocular comorbidities that are likely to affect the test like corneal or macular pathology (such as any corneal opacity or any macular scar). We used the following reliability indices for glaucoma cases: fixation losses (0.2); the fraction was converted to a decimal number form; < 20% for false-positive and false-negative errors.

### Study Procedures 

All the study participants were examined with the HFA (Zeiss/USA) and the VF 2000Ⓡ VR Visual Field Analyzer (MMD/USA) on the same day by the same examiner. We had performed perimetry on all these patients previously at least twice using the HFA; the criteria for fixation losses, false positive, and false negative were based on the acceptable and standard cut-off values.

HFA (Zeiss/USA): The participant sat in a comfortable (rested forehead and chin) position in front of the HFA (Zeiss/USA) bowl in a semi-dark room. The patient was told to look at the central fixation target and click the buzzer whenever the light stimulus was visualized. The lens power and type were provided by the HFA (Zeiss/USA) analyzer in patients with refractive errors. In these cases, wire-rimmed full aperture lenses were used. We tested one eye at a time and the eye which was not being tested was occluded with a patch. We used the Swedish Interactive Thresholding Algorithm (SITA) Standard 24-2 for these cases.

**Figure 1:**
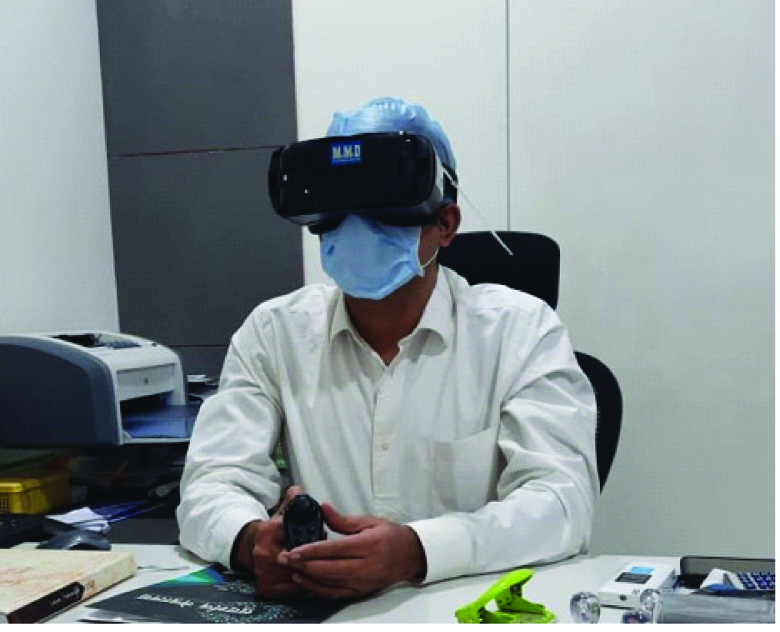
Figure showing the use of the instrument in a participant.

Palm Scan VF2000Ⓡ Virtual reality (VR) Visual Field Analyzer (MMD/USA): Participants wore the VR glasses; these glasses are fitted with a strap and adjusted to avoid any tilt. The participant was told to look at the central fixation target and click the buzzer whenever the light stimulus was visualized. The examiner adjusted the focus using two rotating knobs present on the instrument; this was to correct the refractive errors. The Palm Scan VF 2000Ⓡ VR Visual Field Analyzer (MMD/USA) has an occluder within the system. Thus, even though the eyes were tested alternatively, no external occlusion patch is required [Figure 1a]. All participants underwent the HFA test followed by the Palm Scan VF2000Ⓡ VR Visual Field Analyzer. They were given a rest of 1 hr at least between the tests on these two machines.

**Figure 2:**
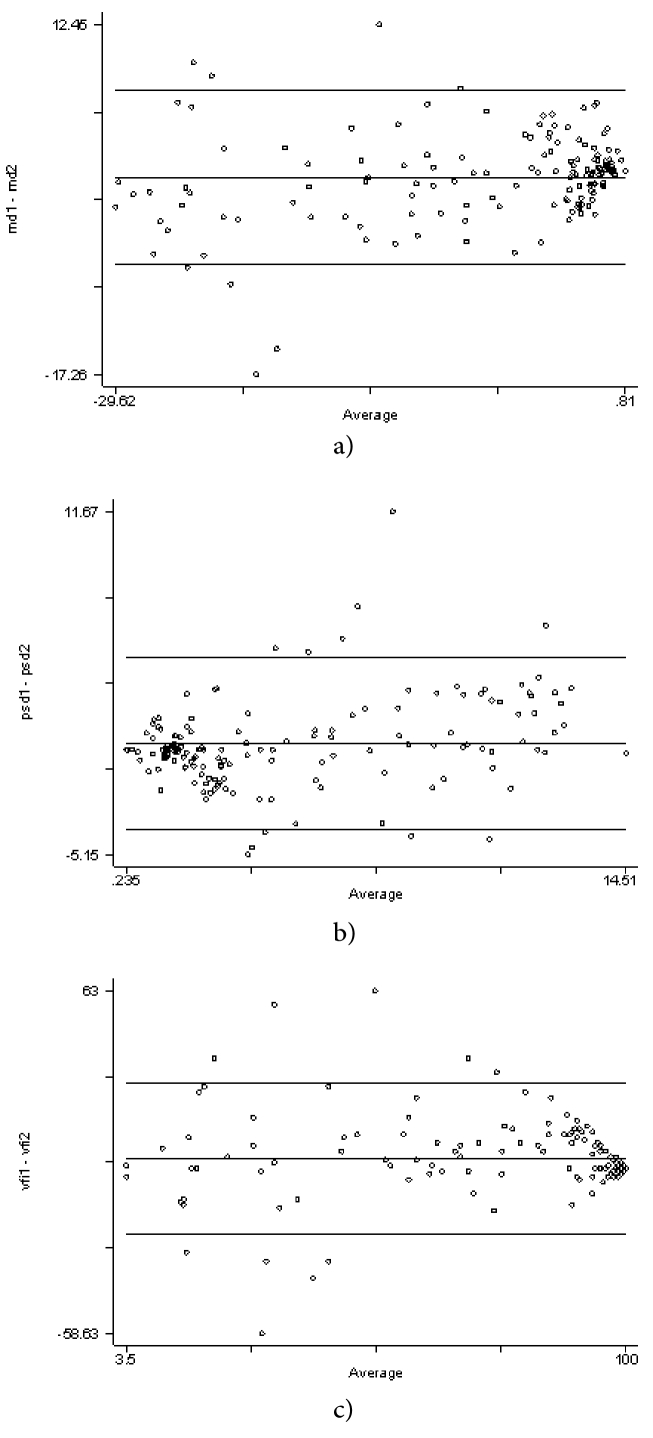
a) Bland Altman plot of the mean deviation values from both the instruments, b) Bland Altman plot of the pattern standard deviation values from both the instruments, c) Bland Altman plot of Visual Field Index values from both the instruments.

We used the central 24-2 threshold test with a stimulus size of three and a presentation time of 200 ms for both these perimeters. The background illumination was 31.5 apostilb for HFA (Zeiss/USA) and 36 decibels for Palm Scan VF 2000Ⓡ VR Visual Field Analyzer (MMD/USA). The software measured fixation losses by the Heijl–Krakau blind spot method. The false positives were events in which the participant responded only to audible stimulus (not visual stimulus) and false negatives were events in which participant failed to respond to supra threshold stimuli.^[10]^

For each test type, we extracted the following data; Mean Deviation (MD), Pattern Standard Deviation (PSD), and Visual Field Index (VFI). Those with an MD of < 6 were classified as mild, with 2–12 as moderate, and with values > 12 as severe glaucoma; this categorization was done according to the Hoddap Classification.^[11]^

### Statistical Analysis

Data were entered in MS Excel (©Microsoft, USA) and analyzed using Stata Version 15.1 (©StataCorp, College Station, Texas, USA). We estimated the means and standard deviation (SD) or median and interquartile range (IQR) for continuous variables, and proportions for categorical variables. The means were compared using *t*-tests and the medians were compared using the Mann–Whitney test. The proportions were compared using the Chi-square test or Fisher's exact test for low expected cell counts.

We estimated the kappa statistic and its 95% confidence interval (CI) as a measure of agreement between the two methods. The diagnostic test properties of Palm Scan VF 2000Ⓡ VR Visual Field Analyzer (MMD/USA) was assessed using sensitivity, specificity, positive predictive value (PPV), and negative predictive value (NPV); HFA (Zeiss/USA) was considered as the gold standard for this analysis. The following analyses were done: (1) comparison of glaucomatous versus non-glaucomatous eyes and (2) severity of glaucoma (mild/moderate/severe) in eyes that were classified as glaucomatous.

We used intraclass correlation coefficient (ICC) as a measure of reliability for continuous variables (MD, PSD, and VFI). These paired values were also visualized using the Bland Altman Plot. A *p*-value of < 0.05 was considered statistically significant.

All procedures performed in studies involving human participants were in accordance with the ethical standards of the institutional and/or national research committee and with the 1964 Helsinki Declaration and its later amendments or comparable ethical standards.

##  RESULTS

The mean age (SD) of individuals was 51.3 (14.9) years. About 62% of participants in the study were males and 38% were females. Of the 86 glaucomatous eyes, 22 (26%) had mild, 18 (21%) had moderate, and 46 (53%) had severe glaucoma (based on the gold standard – HFA [Zeiss/USA]).

### Comparison of Glaucomatous and Non-glaucomatous Eyes

In these analyses, the agreement between Palm Scan VF 2000Ⓡ VR Visual Field Analyzer and HFA for diagnosis of glaucoma was 1.00 (95% CI: 1.00-1.00). According to Palm Scan VF 2000Ⓡ VR Visual Field Analyzer, the proportion for true positives and true negatives was 100% respectively. Thus, the sensitivity and specificity for Palm Scan VF 2000Ⓡ VR Visual Field Analyzer for classifying individuals as glaucoma/non-glaucoma was 100%. The PPV and NPV were both 100%.

### Glaucomatous Eyes

We initially classified these individuals into mild vs moderate/severe glaucoma. The true positive proportion for moderate/severe glaucoma was 92% and the true negative proportion was 86%. Thus, sensitivity and specificity of Palm Scan VF 2000Ⓡ VR Visual Field Analyzer for the detection of moderate/severe glaucoma was 92.2% and 86.4%, respectively. The PPV was 95.2% and the NPV was 79.2%. Detailed estimates and their 95% CI are presented in Table 1.

**Table 1:** Table showing the diagnostic test properties (including the Area Under the Curve) of the Palm Scan VF2000Ⓡ Virtual Reality Visual Field Analyzer in 166 eyes.


	**Estimate**	**95% Confidence Intervals**
**Glaucoma vs non-glaucoma**	
Sensitivity	100%	95.7%-100%
Specificity	100%	95.5%-100%
Positive Predictive Value	100%	95.7%-100%
Negative Predictive Value	100%	95.5%-100%
ROC Area Under the Curve	1.00	1.00-1.00
**Type of glaucoma**	
*** Mild/moderate vs severe***	
Sensitivity	80.4%	66.1%-90.6%
Specificity	90.0%	76.3%-97.2%
Positive Predictive Value	90.2%	76.9%-97.3%
Negative Predictive Value	80.0%	65.4%-90.4%
ROC Area Under the Curve	0.85	0.78-0.93
*** Mild vs moderate/severe***	
Sensitivity	92.2%	82.7%-97.4%
Specificity	86.4%	65.1%-97.1%
Positive Predictive Value	95.2%	86.5%-99.0%
Negative Predictive Value	79.2%	57.8%-92.9%
ROC Area Under the Curve	0.89	0.81-0.97

**Table 2:** Table showing the kappa agreement and 95% confidence intervals between Palm Scan VF2000Ⓡ Virtual Reality Visual Field Analyzer and Humphrey Field Analyzer


	**Kappa estimate**	**95% Confidence Intervals**
**Glaucoma vs non-glaucoma**	1.00	1.00-1.00
**Classification of glaucoma**	
Mild glaucoma	0.76	0.61-0.92
Moderate glaucoma	0.37	0.14-0.60
Severe glaucoma	0.70	0.55-0.85
**Group of glaucoma included**	
Mild or moderate	0.49	0.20-0.78
Moderate or severe	0.43	0.22-0.65
Mild or severe	0.67	0.39-0.95

**Table 3:** Table showing the classification of severity of glaucoma according to Palm Scan VF2000Ⓡ Virtual Reality Visual Field Analyzer and Humphrey Field Analyzer in 86 glaucomatous eyes.


**Humphrey**	**VR Perimetry**			**Total**
	**Mild**	**Moderate**	**Severe**	* *
Mild	1986.36 %	29.09 %	14.55 %	22100 %
Moderate	527.78 %	1055.56 %	316.67 %	18100 %
Severe	00 %	919.57 %	3780.43 %	46100 %
**Total**	2427.91 %	2124.42 %	4147.67 %	86100 %
χ ${}^{2}$ = 72.053; df = 4; Cramer's V = 0.647; Fisher's *p* < 0.001				

We also classified the eyes mild/moderate vs severe glaucoma. The true positive proportion for mild/moderate glaucoma was 90% and the true negative proportion for severe was 80%. The sensitivity of Palm Scan VF 2000Ⓡ VR Visual Field Analyzer for identification of mild/moderate glaucoma was 90.0%, and the specificity was 80.4%. The PPV was 80% and the NPV was 90.2%. We have presented all the diagnostic test properties (estimates and 95% CI) in Table 1.

We also tested the kappa agreement between these two instruments for severity of glaucoma. The overall agreement for severity of glaucoma between these two instruments was 0.63 (95% CI: 0.56-0.78). We have presented kappa values and their 95% CI in Table 2. For classification of glaucoma as moderate or severe, the kappa value was 0.43 (95% CI: 0.22-0.65). The agreement was best for classification of glaucoma as mild or severe (kappa: 0.67-95% CI: 0.39-0.94) [Table 2]. We found that the highest proportion of misclassification was in the moderate group; they were classified as mild (28%) or severe (17%). Furthermore, about 20% of severe cases were misclassified as moderate by the VR Visual Field Analyzer [Table 3].

### Other Parameters

The ICC for MD was 0.96 (95% CI: 0.94-0.97), for PSD was 0.93 (95% CI: 0.92-0.95), and for VFI was 0.92 (95% CI: 0.90-0.95). The Bland Altman plots for these three parameters are presented in Figures 2a–2c. The median (IQR) fixation loss in the HFA group was 0.45 (0-13.3) and in the Palm Scan VF 2000Ⓡ VR Visual Field Analyzer group was 0 (0-18.2); the difference was not statistically significant (*p* = 0.89). Similarly, the median (IQR) difference for false negative responses was not significantly different in both these methods (HFA (Zeiss/USA): 0 [0, 6] vs Palm Scan VF 2000Ⓡ VR Visual Field Analyzer (MMD/USA): 0 [0, 18]; *p* = 0.07). However, we found the median (IQR) false positives were significantly higher in the HFA (0 [0, 2]) compared with that of the Palm Scan VF 2000Ⓡ VR Visual Field Analyzer (0 [0, 0]); the difference was statistically significant (*p* = 0.0003).

##  DISCUSSION

This study showed that Palm Scan VF2000Ⓡ VR Visual Field Analyzer had a perfect agreement with HFA for the detection of glaucoma. The sensitivity and PPV for identifying glaucoma were 100%; however, the sensitivity, specificity, PPV, and NPV was lower for the severity of glaucoma. The agreement was best for the classification of glaucoma as mild or severe; however, the agreement was not good for classification cut-off at mild or moderate, and moderate or severe.

Glaucoma may go unnoticed in the early stages as it starts with loss of peripheral vision. The patient may not realize the loss and hence may not seek any medical advice.^[12]^ Hence, it is important to have screening tools for this disease so that patients in initial stages can be identified because in glaucoma, optic nerve damage is irreversible and it may progress in most cases without appropriate treatment.^[13]^ As seen in our study, the VR Visual Field Analyzer had perfect agreement^[14]^ with the HFA for classification of eyes as glaucomatous or non-glaucomatous; the diagnostic test properties were also good. In fact, the sensitivity and specificity observed in our study was better compared with that of the optical coherence tomography (OCT) for classification of eyes as glaucomatous or non-glaucomatous.^[15]^ Tpaskis and colleagues, and Mees and coworkers found an excellent correlation between these two methods in detecting glaucoma.^[7,16]^ The main advantage of the HFA is the current gold standard. However, the disadvantages are that it cannot be used in community screenings due to the difficulty in transportation of the instrument or for patients who are unable to sit or are immobile (due to any reason such as post-surgery). The main advantage of the VR perimetry is that it can be used for community- and clinic-based screenings. It can also be used with patients with back pain who have difficulty to sit for perimetry or those who are immobile or confined to the bed.^[6,7]^ However, it cannot be used to identify the severity of glaucoma and hence is not very useful in the management of the condition in the present form.

It has been suggested that due to the subjectivity in visual filed testing, the variability in examination recorded is likely to be higher when the damage is greater.^[7,17,18]^ In general, portable and/or tablet based, or online perimeters have shown to be reliable and assess the visual fields fairly accurately,^[8,19,20,21,22]^ however, a recently published report found that a VR head-mounted device did not identify the deficits reliably.^[16]^ As seen in our study, the agreement between these two instruments was best when the glaucoma was classified as either mild or severe. Furthermore, we did find that a large proportion of moderate glaucoma cases were misclassified as mild. Thus, the present algorithm is not able to classify the glaucoma appropriately.

The study was conducted in a clinic-based setting; this is a more controlled setting with better co-operation by patients. Hence, we may have overestimated some of the diagnostic test properties. For example, patients who came to the clinic were more likely to be aware of glaucoma and its importance for vision. Thus, they are more likely to adhere to all the instructions during these tests. This may influence the test results. It will be appropriate to test the properties of this instrument in community settings as well. Although, we would like to suggest the use of this instrument as a screening tool for glaucoma, a community-based study will provide additional evidence to this effect.

This study is an important contribution to the literature. We went beyond the glaucoma/non-glaucoma differentiation and evaluated the instrument for classification of glaucoma. We did find that the instrument in its current form is able to differentiate between glaucomatous and non-glaucomatous eyes. However, among patients with glaucoma, the instrument is not able to correctly classify the stage of glaucoma. Particularly, moderate glaucoma is more likely to be misclassified as mild or severe. Hence, it cannot replace the HFA in clinical settings in its current form for the management of glaucoma. The algorithm needs to be refined to account for this discrepancy. The instrument may be used in screening of individuals for the presence/absence of glaucoma in community settings, health camps, and clinical practices. It is also useful for those patients who are unable to come to the examination room or sit in the examination chair due to back problems/surgeries, old age, neck problems, and disabilities. However, based on the evidence generated from this study, in the current form, the instrument may only be used as a screening tool for identification of glaucoma.

##  Financial Support and Sponsorship

Nil.

##  Conflicts of Interest

There are no conflicts of interest.
